# Investigation of the High-Temperature and Rheological Properties for Asphalt Sealant Modified by SBS and Rubber Crumb

**DOI:** 10.3390/polym14132558

**Published:** 2022-06-23

**Authors:** Yafeng Gong, Shuzheng Wu, Yuwei Zhang, Yunze Pang, Yulin Ma

**Affiliations:** College of Transportation, Jilin University, Changchun 130025, China; gongyf@jlu.edu.cn (Y.G.); wusz20@mails.jlu.edu.cn (S.W.); pangyz19@mails.jlu.edu.cn (Y.P.); ylma18@mails.jlu.edu.cn (Y.M.)

**Keywords:** sealant, SBS, rubber crumb, high-temperature performance, permanent deformation resistance

## Abstract

Crack sealing is an important measure for pavement maintenance. Hot-poured crack sealant is the most utilized material for crack sealing. However, its poor high-temperature and rheological properties seriously weaken the mechanical properties of repaired pavement. Thus, to overcome the disadvantage of the poor high-temperature and rheological properties of sealant, styrene–butadiene–styrene (SBS) and rubber crumb (CR) were utilized for modifying the asphalt-based sealants. Softening point tests, temperature tests, frequency scan tests, and multiple stress creep recovery tests (MSCR) were conducted to evaluate the high-temperature and rheological properties of the modified sealant. Additionally, the influence of SBS and CR on the high-temperature performance of the modified sealant was quantitatively analyzed by the grey relational analysis method. The results reveal that the SBS has a greater enhancement effect on the high-temperature performance of sealant than CR. Increasing the SBS and CR content in the sealant could enhance the sealant’s high-temperature performance, stiffness, and elasticity. Compared with asphalt-based sealant and one-component modified asphalt-based sealant, SBS/CR-modified asphalt sealant has greater viscosity and higher temperature deformation resistance. Additionally, SBS can increase the stress level of the sealant, thereby enhancing the resistance of the sealant to permanent deformation.

## 1. Introduction

Asphalt pavement is widely used in road engineering for its advantageous properties, which include short construction period, low noise, safety, comfortable driving, recyclability, etc. In recent years, with the rapid increase in traffic volume and vehicle load, serious asphalt pavement damage has occurred, and road maintenance measures have gradually become key to guaranteeing service performance [1,2]. Among many types of pavement damage, pavement cracking is one of the most common forms of damage, not only destroying the integrity of the pavement, but also leading to the infiltration of water, softening the base, weakening the bearing capacity of the base, and causing structural damage to the road [3,4,5]. Most of the pollution caused by asphalt pavement is secondary environmental pollution caused by pavement damage resulting from poor asphalt durability. Using asphalt to cover the earth as a road surface was once considered to be contrary to environmental friendliness. However, the environmental pollution caused by refurbishment due to road damage will undoubtedly lead to more serious environmental degradation. Engineering practice shows that crack sealing is an effective way of dealing with cracking-type damage to pavement [6,7]. Therefore, increasing attention is being paid to research into sealing materials, and various types of sealants have emerged in accordance with the requirements of the times [8]. Among these, heated asphalt-based sealant has been widely used in road maintenance projects for its outstanding crack sealing effect and low price [9,10].

However, the sealant material left on the surface after filling the road cracks will be directly exposed to the natural environment and constantly exposed to sunlight. On-site investigations have shown that the maximum temperature of asphalt pavement exposed to direct sunlight can exceed 60 °C. Continuous high temperature is likely to cause sealant aging, premature decline in mechanical properties, and a reduction in the bonding force with the road, meaning that the asphalt can be easily carried away by vehicles [11]. Therefore, it is important to improve the high-temperature stability of the sealant material [12,13].

Asphalt-based sealant materials are mainly composed of matrix asphalt, rubber powder, various polymers, softeners, fillers, etc., and improve the performance of asphalt sealants/mastics by introducing different waste/virgin polymers. There are many kinds of polymer-modified asphalt, the most common of which are rubber-modified asphalt using rubber powder as modifier. Thermoplastic-rubber-modified asphalt includes modifications with styrene-butadiene-styrene (SBS), styrene-isoprene (SIS), styrene-polyethylene-butadiene-styrene (SEBS), and other block copolymers. Resin-based asphalt modifications include the addition of polyethylene (PE) and ethylene-vinyl acetate copolymer (EVA) [14]. For example, Rosa Veropalumbo and Russo, F. et al. [15,16,17] have been working on the incorporation of polymer-functional plastic waste into asphalt mixtures to improve the performance of pavement layers. Since the asphalt-based sealant material prepared with a single modifier has disadvantages in terms of performance, and the single modifier is expensive, some low-cost modifiers need to be used to reduce costs; therefore, composite modification has been developed and applied [14]. With advances in technology, SBS has gradually replaced other types of polymers due to its superior high- and low-temperature properties. In view of the excellent anti-deformation and anti-cracking properties of rubber-modified asphalt, this paper uses CR/SBS to modify asphalt in order to study the properties of the sealant material. 

A huge amount of waste rubber tires is generated globally every year. The reasonable disposal of the ever-increasing number of waste rubber tires is a global concern, and the tires could cause serious harm to the ecological environment if disposed of improperly. Some of these waste tires are eventually landfilled or incinerated, causing pollution to either land resources or air, and posing a severe threat to the environment. In recent years, scholars have begun to focus on the addition of waste rubber tires, physically ground into a granular state, into construction materials to prepare modified asphalt or concrete, thus realizing the recycling of waste rubber and opening a novel route for the treatment of waste rubber products. In the field of pavement material preparation, rubber crumb (CR)-modified asphalt presents good anti-deformation and anti-cracking ability [18,19,20]. However, the compatibility between CR and matrix asphalt is poor, and the high-temperature resistance of CR is not stable, which shortens the service life of CR-modified asphalt [21,22]. In addition, in the preparation process of CR-modified asphalt, CR expands due to the absorbance the light components (aromatic and saturated) of asphalt, increasing the viscosity of the asphalt and therefore affecting the fluidity of the sealant material made from CR-modified asphalt [23,24,25]. Therefore, in the production of CR-modified asphalt sealant, the proportion of added rubber, mixing temperature, and the performance of the matrix asphalt have a crucial impact on its high-temperature resistance and other properties [26,27].

Styrene-butadiene-styrene (SBS) is currently the most widely used asphalt modifier. It possesses a multiphase structure and is mainly composed of a butadiene segment and a styrene segment. SBS melts when the ambient temperature is between the glass transition temperatures of styrene and butadiene [28,29]. SBS-modified asphalt exhibits both high-temperature resistance and high elasticity, which is due to the cross-linking of SBS inside the asphalt to create a stable three-dimensional network structure [30]. Therefore, utilizing SBS-modified asphalt as the sealant for filling pavement cracks will significantly improve its high-temperature resistance. However, the cost of SBS-modified asphalt is relatively high, so the proportion of SBS must be controlled when preparing sealant in order to reduce the engineering cost, while at the same time guaranteeing good service performance (such as high-temperature properties) [31]. Thus, some researchers have thought of combining SBS and CR to modify asphalt materials so as to improve their high-temperature resistance while reducing their cost [32].

In summary, although much literature has reported the high-temperature resistance properties of CR/SBS-modified asphalt, as a sealant material for cracked pavements, the evaluation indices for the high-temperature performance of CR/SBS-modified asphalt sealant are incomplete. Further performance evaluation is needed to verify whether it meets the requirements of the Chinese national standard, making it suitable for being adopted in actual road maintenance projects [33,34]. Meanwhile, the asphalt sealant is often subjected to continuously repeating vehicle load; therefore, its real mechanical response needs to be further evaluated on the basis of the rheological performance of the sealant under dynamic load [35,36].

This study is dedicated to preparing an eco-friendly asphalt sealant material for pavement maintenance engineering that possesses excellent high-temperature stability and anti-deformation ability through the addition of waste rubber and SBS, which is expected to expand the application of waste rubber in pavement maintenance engineering, representing a more sustainable utilization of waste rubber products, thus protecting the ecological environment. In addition, softening point, complex shear modulus, phase angle, mean non-recoverable creep modulus, and other indicators are used to systematically evaluate the high-temperature properties and rheological properties of the modified asphalt sealants. Firstly, the enhancement effect of SBS and CR on the high-temperature stability of asphalt was evaluated separately. Secondly, the differences in the high-temperature performances of the SBS/CR-modified asphalt sealant, unmodified asphalt sealant (UAS), SBS-modified asphalt sealant, and CR-modified asphalt sealant were evaluated. Subsequently, the high-temperature stability and anti-deformation ability of the SBS/CR asphalt sealant was evaluated on the basis of softening point tests, temperature scan tests, frequency scan tests, MSCR, and dynamic shear rheology tests (DSR). The master curve of modulus for the SBS/CR sealant was established, and the viscoelastic properties of the SBS/CR sealant were evaluated over a wide frequency range.

## 2. Materials and Methods

### 2.1. Raw Materials

Donghai Brand 90# road petroleum asphalt, produced by China Petrochemical Corporation in Beijing, China, was used as the matrix asphalt for the pavement sealant. The test process was determined with reference to the standard test methods of Asphalt and asphalt mixture test regulations for highway engineering (JTG E20-2011) [37]. The technical indices of the matrix asphalt are provided in Table 1. The selected SBS (grade 4402), produced by China Petrochemical Corporation, was a star-shaped structure, with a styrene content of 30 wt%. CR was purchased from Shaanxi Hongrui Rubber Co., Ltd. in Xi’an, China, with a particle size of 0.3 mm; the technical indices are listed in Table 2.

### 2.2. Mixture and Preparation for the CR- and SBS-Compound-Modified Asphalt Sealant

The preparation technique for the modified asphalt sealant is as follows: (1) heat 500 g of 90# matrix asphalt to a fluid state at 140 °C; (2) add SBS to the sample and stir at a speed of 800 r/min for 30 min on a high-speed mixer at 170 °C; (3) cut the sample at a speed of 5000 r/min for 45 min on a high-speed shearing machine at 170 °C; and (4) add CR to the sample and stir at a speed of 800 r/min for 1 h on a high-speed mixer at 170 °C to produce the modified asphalt sealant.

Mixing excessive CR into the asphalt will lead to an increase in the viscosity of the modified asphalt sealant, which will result in asphalt segregation [38]. This is because the swelling rate of CR does not increase infinitely, and reaches a peak when the content is around 20% [39]. The softening point of the modified asphalt sealant depends mainly on the amount of dispersed SBS and the perfection of the star-shaped structure. When the content of SBS is about 4% to 6%, a relatively perfect network structure will be formed in asphalt. Therefore, the use of 4% to 6% SBS in modified asphalt production is most appropriate, in accordance with Chinese Standard (JT/T 740-2015) [40]. The research presented in this paper also take the content of star-shaped SBS as a variable for conducting research. Hence, 12 groups of sealants based on CR- and SBS-compound-modified asphalt sealants were prepared in this study; 3 samples were prepared for each group, and experimental error was eliminated by averaging the results. The mix proportions are listed in Table 3.

### 2.3. Test Methods

#### 2.3.1. Softening Point Test

Asphalt softening point is an important indicator of asphalt sealant performance, and is usually used to measure the temperature sensitivity of asphalt sealant. In this study, the softening point values of all groups of the sealant samples were tested according to the test procedure of the Chinese Standard (JTG E20-2011) [37]. The softening point experiments were performed using a DF-10 computer automatic softening point instrument produced by Nanjing Dongyong Shenfu Technology Co., Ltd. in Nanjing, China. Note that the softening point tests for the sealant samples were carried out using the ring and ball softening point method. The sealant samples were cured in the specified curing environment, in line with the test requirements. Then, the sealant samples were heated at a rate of 5 °C/min in the cup, and the heating and cooling temperatures were recorded. The average value of the temperature is taken as the softening point test result for each sealant sample.

#### 2.3.2. Temperature Sweep Test

A Dynamic Shear Rheometer (DSR) produced by Anton Paar Co., Ltd. in Graz, Austria (Model: SmartPave 102) was utilized in this study to conduct temperature sweep experiments on modified asphalt sealant. The test results were used to reflect the rheological behavior of the asphalt-based material at medium and high temperature. The temperature range of the test was 34~94 °C, and the temperature gradient was 6 °C, as well as having an angular frequency of 10 rad/s. The complex shear modulus (G*) and phase angle (*δ*) were tested, and the change rule of the viscoelastic parameters was evaluated, which was used to determine the temperature dependency of the samples.

#### 2.3.3. Muti-Stress Creep and Recovery Test

The muti-stress creep and recovery (MSCR) tests were used to measure the deformation value of asphalt-based material under different constant stress and the creep deformation recovery values after the disappearance of stress. The stress control approach was used in the repeated stress creep recovery experiments. The test process can be divided into the creeping stage, with a constant stress loading time of 1 s, and the recovery stage, with a zero-stress no-load time of 9 s under each cycle at 64 °C. For each experiment, 30 stress cycles were carried out. The adopted stress for cycles 0~20 was 0.1 kPa, and for cycles 21~30 it was 3.2 kPa. Nevertheless, the test results for cycles 0~10 were only used to adjust the properties of the asphalt sealant, and were not used for analysis. During the test, stress and strain were recorded at a frequency of 0.1 s during the creep phase and at a frequency of at least 0.45 s during the recovery phase.

#### 2.3.4. Frequency Sweep Test

**Figure 1:**
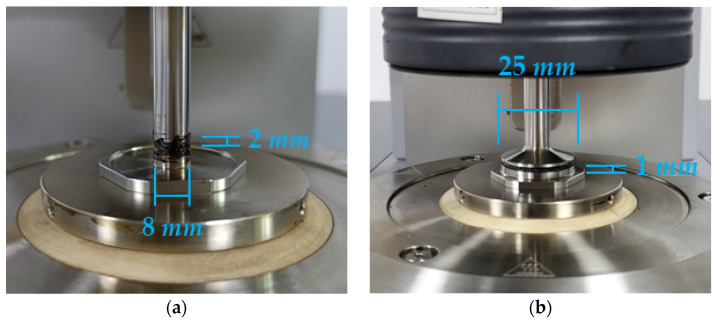
Parallel plate size and sealant sample size in DSR at different temperatures. (**a**) 30 °C and 40 °C; (**b**) 50 °C, 60 °C and 70 °C.

To study the mechanical properties of modified asphalt sealant materials under different loading frequencies, frequency sweep tests for different sealant samples were performed at a test frequency of 0.1~100 Hz at different temperatures (30 °C, 40 °C, 50 °C, 60 °C, and 70 °C). The parallel plate’s diameter was 8 mm, and the thickness values of the sealant samples were 2 mm at 30 °C and 40 °C, while the parallel plate’s diameter was 25 mm and the thickness values of the sealant samples were 1 mm at 50 °C, 60 °C, and 70 °C (as shown in Figure 1). Before the frequency sweep test, the parallel plates were heated to 70 °C in order to avoid damage to the sealant samples when removing excess asphalt and achieve a good connection between the asphalt samples and the parallel plates.

## 3. Results and Discussion

### 3.1. Softening Point Results and Grey Correlation Analysis

**Figure 2:**
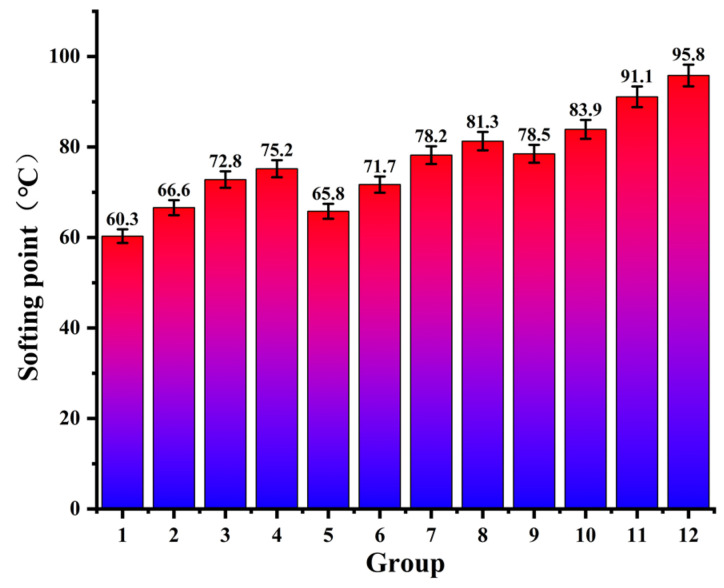
The results of the softening point test.

This study used softening point values to assess the high-temperature performance of modified asphalt sealant samples (as shown in Figure 2), and the effect of CR and SBS on high-temperature performance for the modified asphalt sealant was investigated.

As shown in Figure 2, the softening point of modified asphalt sealant increases with increasing CR and SBS content, which indicates that CR and SBS can significantly enhance the high-temperature performance of asphalt sealant [39]. With reference to the softening point regulations for hot-poured sealants for pavement (JT/T 740-2015) [40], the required minimum softening point for sealant is 70 °C. Compared with the matrix asphalt, the softening points of Group 1, Group 2, and Group 5 were significantly improved, but were still not able to meet the requirements of the sealant. This shows that the sealants these contents were not able to achieve the best performance, which could be achieved by increasing the modifier content in order to meet performance requirements [41]. When the SBS content was 20% and the CR content was 7% (Group 12), the softening point of the modified asphalt sealant reached 95.8 °C, which is 58.9% higher than that of Group 1. When the SBS content in the modified asphalt sealant was constant, the softening point value increased by 12.9% on average with a 5% increase in CR content. Similarly, when the CR content was constant, the softening point value increased by 7.3% on average with a 2% increase in SBS content [42]. Furthermore, it can be seen from the test results that the modification effect of SBS per unit content is about 1.4 times that of CR. Intuitively, the increase in unit SBS content had a greater impact on the softening point of the asphalt sealant than CR. 

Nevertheless, the analysis of the basic data cannot be used to accurately measure the degree of correlation between the modifier and the high-temperature performance of the modified asphalt sealant. Therefore, the grey correlation degree was used to further analyze the basic data. Grey correlation analysis is an academic analysis method for multi-factor degrees of correlation and the comparison of data development trends [43,44], and has been successfully applied in agronomy, medicine, management, and business [45,46]. In this paper, the softening point was selected as the reference sequence, and the correlation degree between CR, SBS content, and high-temperature performance in a single-component modified asphalt sealant was analyzed using the grey correlation analysis method. 

According to the theory of grey relational analysis, it is first necessary to determine the comparison sequence and the reference sequence:

The comparison sequence is written as Equation (1):

(1) $$X_{1}^{\prime },X_{2}^{\prime },\ldots X_{n}^{\prime }=\left(\begin{array}{cccc} X_{1}^{\prime }\left(1\right) & X_{2}^{\prime }\left(1\right) & \cdots & X_{n}^{\prime }\left(1\right)\\ X_{1}^{\prime }\left(2\right) & X_{2}^{\prime }\left(2\right) & \cdots & X_{n}^{\prime }\left(2\right)\\ \vdots & \vdots & \vdots & \vdots \\ X_{1}^{\prime }\left(m\right) & X_{2}^{\prime }\left(m\right) & \cdots & X_{n}^{\prime }\left(m\right) \end{array}\right)$$

The reference sequence is an ideal comparison standard in theory. The optimal value (or worst value) of each index can be used to form the reference sequence, and other reference values can also be selected according to the purposes of the evaluation. The reference sequence is written as: $X_{0}^{\prime }=X_{0}^{\prime }\left(1\right),X_{0}^{\prime }\left(2\right),\ldots X_{0}^{\prime }\left(m\right)$.

The index data are dimensionless for easy comparison. In this paper, the difference method is used to obtain the data sequence matrix. The absolute differences between each evaluated object index sequence (comparison sequence) and the corresponding element of the reference sequence are calculated one by one. Then, the correlation coefficient between each comparison sequence and the corresponding element of the reference sequence is calculated according to Equation (2):

(2) $$\zeta_{i}\left(k\right)=\frac{\begin{array}{c} min\\ i \end{array}\begin{array}{c} \;min\\ k \end{array}\left|X_{0}\left(k\right)-X_{1}\left(k\right)\right|+\rho \cdot \begin{array}{c} min\\ i \end{array}\begin{array}{c} \;min\\ k \end{array}\left|X_{0}\left(k\right)-X_{1}\left(k\right)\right|}{\left|X_{0}\left(k\right)-X_{1}\left(k\right)\right|+\rho \cdot \begin{array}{c} min\\ i \end{array}\begin{array}{c} \;min\\ k \end{array}\left|X_{0}\left(k\right)-X_{1}\left(k\right)\right|}\;\;\;\;\;\;\;\;\;\;\;k=1,2,\ldots m$$

where ρ is the resolution coefficient, 0 < ρ < 1. Smaller values of ρ indicate a larger difference between the correlation coefficients, and stronger discrimination ability. Usually, ρ is taken as 0.5.

For each evaluation object, the mean value of the correlation coefficient between each index and the corresponding element of the reference sequence is calculated to reflect the relationship between each evaluation object and the reference sequence; this is referred to as the correlation sequence, and is determined using Equation (3) [44]:

(3) $$R=\frac{1}{m}\overset{m}{\underset{k}{\sum }}\zeta_{i}\left(k\right)$$

The difference sequences for each factor were calculated, and the results are listed in Table 4. The grey correlation coefficients under different contents were calculated, and the results are listed in Table 5.

The quantitative characterization of the effect of SBS and CR on high-temperature performance was realized through correlation analysis. It can be seen from Table 5 from the correlation coefficients between the modifiers (CR and SBS), as well as the order of the grey relation coefficients from high to low, that R2 > R1. The correlation between SBS and asphalt sealant softening point is 1.5 times that of CR, which also confirms the previous conclusions. The correlation sequence results show that, compared with CR, the content of SBS has a greater correlation with the high-temperature performance of the modified asphalt sealant.

Most previous studies have concluded that both CR and SBS are able to improve the softening point of asphalt sealants to a certain extent [4,20]. However, there have been few studies on the softening point improvement efficiency of SBS and CR in CR/SBS-composite-modified asphalt. This paper confirms the dominant role of SBS in affecting the softening point of sealants through grey correlation. SBS is the most significant factor affecting the softening point in the CR/SBS-modified asphalt sealant. Therefore, when preparing environmental protection sealants, we should not only consider the addition of waste/virgin polymers, but also ensure an adequate content of SBS, in order to meet the high-temperature performance requirements.

Both CR and SBS have a significant effect on the softening point of the modified asphalt sealant. The changing trend of the softening point shows that 20% CR generally has higher conventional high-temperature performance. Hu M et al. [39] also reported that once the content of rubber powder has reached 20%, the viscosity of the dispersion medium is no longer significantly improved by increasing content and mesh number. To improve creep recovery ability and rheological properties under dynamic load, once the requirements of conventional high-temperature performance had been satisfied, SBS was added. The grey correlation analysis results show that SBS has a greater impact on the softening point of the modified asphalt sealant (conventional high-temperature performance). However, it is still necessary to investigate whether higher SBS content results in more beneficial rheological performance in the modified asphalt sealant under high-temperature conditions in order to determine the optimal amount of added SBS. In the experiments that follow in this paper, the rheological properties are studied by controlling the gradient of SBS content for the asphalt sealant. Under the premise of satisfying a certain high-temperature performance (20% CR), the rheology of the modified asphalt sealant can be optimized to the greatest extent so that the performance can be improved more comprehensively while taking the project cost into account.

The three groups with the best softening point performance in the orthogonal experiment were selected: Group 10, Group 11, and Group 12. Therefore, the temperature sweep test was carried out on Group 10, Group 11, and Group 12. Three control groups were determined: unmodified asphalt sealant (marked as Group a), single-component modified asphalt with 7% SBS content (marked as Group b), and single-component modified asphalt with 20% CR content (marked as Group c). The MSCR test and frequency sweep test were carried out together for Group 10, Group 11, and Group 12. The high-temperature performance of MSCR and the viscoelastic mechanical properties of composite-modified asphalt sealant were evaluated.

### 3.2. Anti-Deformation Ability

**Figure 3:**
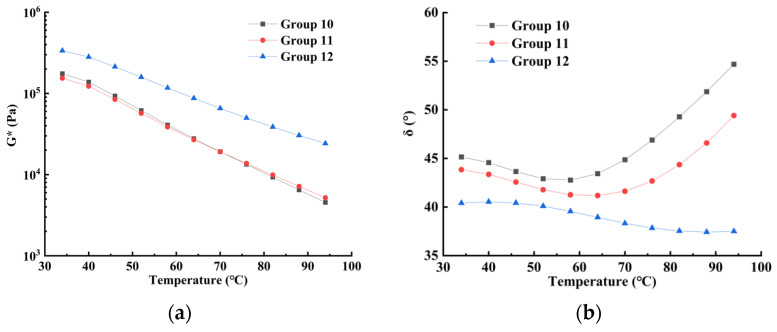
The temperature scanning test results. (**a**) The complex shear modulus; (**b**) the phase angle.

It is well known that the viscoelastic properties of modified asphalt sealant are sensitive to temperature. Therefore, it is necessary to evaluate the sensitivity of the viscoelastic properties of SBS/CR-modified asphalt sealant to verify whether this new asphalt sealant is able to meet the mechanical performance requirements of future road maintenance engineering applications. Hence, a temperature scanning test was carried out on the modified asphalt sealant (Group 10, Group 11, and Group 12) to evaluate their temperature sensitivity. In the temperature sweep test, the complex shear modulus (G*) can be used to evaluate the anti-deformation ability of asphalt sealant. The higher the composite shear modulus, the greater the high-temperature deformation ability of the asphalt sealant. In addition, the viscoelastic characteristics of asphalt sealant could be assessed by the phase angle (*δ*). The asphalt sealant becomes more pliable with decreasing phase angle, which means that the asphalt sealant has stronger high-temperature resistance [47,48]. In this study, the variation in the parameters G* and *δ* with modifier content (CR and SBS) was analyzed at different temperatures. Parallel experiments were performed for three samples in this experiment, and the maximum standard deviation was calculated to be 2.7345 by filtering and integrating the data. The average value of the data points was calculated, and the test results are shown in Figure 3.

On the basis of the test results presented in Figure 3a, the complex shear modulus G* of the modified asphalt sealant shows a decreasing trend with increasing temperature. In the semi-logarithmic coordinates, the regularity of the change of G* with temperature approximates a linear functional relationship. Furthermore, the curves of Group 10 and Group 11 have similar trends and values, indicating that the two sealants have the same anti-deformation ability within the temperature range 34~94 °C. The complex shear modulus of the three sealants is about 1.75 × 10^5^ Pa at 34 °C, while the complex shear modulus of the Group 12 sealant decreases to 2.4 × 10^4^ Pa, and the modulus values of the Group 10 and Group 11 sealants decrease to 4.5 × 10^3^~5.2 × 10^3^ Pa at 94 °C. Compared with Group 10 and Group 11, the complex modulus of Group 12 has a smaller variation in amplitude with angular frequency, which also means that its temperature sensitivity is lower. These experimental results are consistent with the results reported by Liang M. [22].

In addition, the phase angle of the Group 10 and Group 11 modified asphalt sealants first decreased and then increased with increasing temperature, which indicates that the viscous and elastic components of the two sealants change continuously at different temperatures. When the temperature is lower than 60 °C, the proportion of elastic components in the modified asphalt sealants increases with increasing temperature, which implies that the deformation resistance of the modified material gradually increases. When the temperature is higher than 60 °C, the proportion of viscous components in the modified asphalt sealants increases gradually with increasing temperature, revealing that the deformation resistance of the modified asphalt sealant decreases. Moreover, the change in the phase angle of the Group 12 sealant is small, and the phase angle is less than 45° within the test temperature range, meaning that modified asphalt sealants with high modifier contents (CR and SBS) have high elasticity and low temperature sensitivity. The temperature change has a small impact on the proportion of the viscoelastic components.

From the complex modulus and phase angle obtained from the temperature sweep test, it can be seen that the complex modulus of Group 12 is generally higher than that of Group 10 and Group 11, although this shows that its stiffness and resistance to deformation are higher. However, its phase angle is always lower than 45° in the test temperature range, and even decreases with increasing temperature, meaning that its viscoelasticity fails to reach equilibrium. After repairing the crack, the adhesion to the original crack wall is insufficient, and the toughness is insufficient due to its excessive hardness. The modulus values of Group 10 and Group 11 are similar. In the high-temperature range of 60~94 °C, Group 11 shows better elastic behavior than Group 10 and has stronger resistance to foreign body embedding. Therefore, when the content of CR is 20%, when preparing SBS/CR-modified asphalt sealant, the addition of 5% SBS provides the best deformation resistance and elastic toughness for the modified asphalt sealant.

### 3.3. High-Temperature Performance on the Basis of MSCR

The deformation of asphalt sealant is a dynamic process caused by the combined effect of dynamic load and temperature. The MSCR test is able to accuratley describe the high-temperature deformation resistance of asphalt sealant under the synergistic action of dynamic load and temperature [49]. MSCR tests were conducted on unmodified asphalt sealant, SBS-modified asphalt sealant, CR-modified asphalt sealant, and SBS/CR-modified asphalt sealant. The average recovery rate ($\bar{R}$) and the average non-recoverable creep modulus ($\bar{J_{nr}}$) at different creep stress levels were used to evaluate the capacity of asphalt sealant to withstand deformation on a permanent basis under various creep stress levels. The calculation method was as shown in Equations (4) and (5):

(4) $$R=\frac{\varepsilon_{c}-\varepsilon_{0}}{\varepsilon_{r}-\varepsilon_{0}};\bar{R}=\overset{10}{\underset{N=1}{\sum }}\frac{R\left(\tau ,N\right)}{10}$$

(5) $$J_{nr}=\frac{\varepsilon_{r}-\varepsilon_{0}}{\tau };\bar{J_{nr}}=\overset{10}{\underset{N=1}{\sum }}\frac{J_{nr}\left(\tau ,N\right)}{10}$$

where τ (kPa) is the creep stress corresponding to each loading period; $\bar{R}$ is the average recovery rate; $\bar{J_{nr}}$ (kPa^−1^) is the average unrecoverable creep modulus.

**Figure 4:**
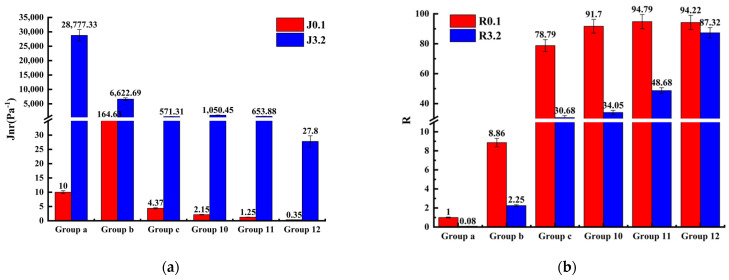
MSCR test results (test temperature: 64 °C). (**a**) The average non-recoverable creep modulus; (**b**) the average recovery rate.

The average recovery rate represents the elastic response of asphalt sealant, while the average unrecoverable creep modulus represents the viscous deformation of asphalt sealant. As shown in Figure 4, the average unrecoverable creep modulus of SBS/CR-modified asphalt sealants was lower than that of the CR-modified asphalt sealant and the SBS-modified asphalt sealant when the degree of stress was 0.1 kPa and the test temperature was 64 °C, while the SBS/CR-modified asphalt sealant’s average recovery rate was comparable to that of the CR-modified asphalt sealant and significantly superior to that of the SBS-modified asphalt sealant. This indicates that modification with SBS and CR enhanced the stiffness and elasticity of the asphalt sealant, thus increasing its deformation resistance.

The average unrecoverable creep modulus of asphalt increased and the average recovery rate dropped when the degree of stress was increased from 0.1 kPa to 3.2 kPa at 64 °C, indicating that the deformation resistance of the asphalt sealant decreases under high load. Hence, high traffic volumes will increase the risk of the asphalt pavement breaking. However, when the percent of SBS in the modified asphalt sealant was increased, the growth rate of the unrecoverable creep modulus decreased significantly, and the reduction range of the average recovery rate slowed down, indicating that the ability of the modified asphalt sealant to withstand permanent deformation at high stress levels increases with increasing SBS content.

Ren S.S. et al. [50] calculated the average non-recoverable creep modulus and the average recovery rate. Accurate quantitative evaluation of the effect on rutting resistance of modifying asphalt with CR/SBS was performed. Low $J_{nr}$ with high *R*-value CR/SBS-modified asphalt outperformed the unmodified asphalt at both stress levels, indicating that the presence of elastic response in the modified asphalt makes it less sensitive to rutting and permanent deformation. This is due to the interaction of rubber powder and SBS mesh in asphalt, which is beneficial for hindering the accumulation of permanent strain. In this section, not only the above conclusions are verified, but 20% CR and 7% SBS-modified asphalt sealant were demonstrated to offer the best stiffness and elasticity, as well as excellent resistance to deformation.

### 3.4. Viscoelastic Mechanical Properties and CAM Model Fitting

**Figure 5:**
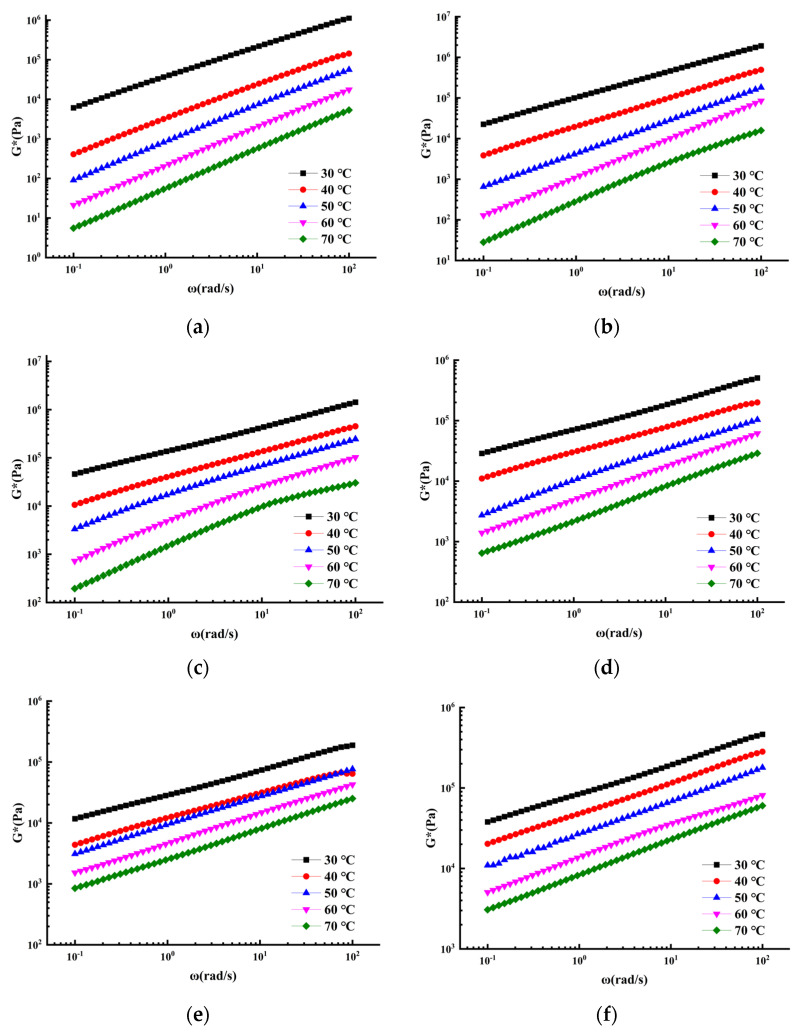
Frequency scanning test results. (**a**) Group a, (**b**) Group b, (**c**) Group c, (**d**) Group 10, (**e**) Group 11, (**f**) Group 12.

As a viscoelastic material, the properties of asphalt vary with temperature and loading time, which has a great impact on its uses in road engineering [1,9,27]. The softening point index described in Section 3.1 can only be used to carry out a macro evaluation of the high-temperature resistance of asphalt sealant, and cannot achieve an accurate description of the microstructure of the asphalt sealant [22]. Consequently, the frequency scanning test was used to evaluate the viscoelastic behavior of asphalt sealant from a microscopic point of view. Similarly, parallel experiments were performed on three samples in this experiment, and the average value of the data points was calculated. Figure 5 shows the average experimental results of the frequency scanning of Group a, Group b, Group c, Group 10, Group 11, and Group 12. The composite shear modulus of the six sealants increased with increasing frequency and decreased with increasing temperature. This phenomenon is more obvious in the unmodified asphalt sealant, which demonstrated poor deformation resistance at high temperature [34].

As a kind of viscoelastic material, the change in the mechanical properties of asphalt abides by the principle of time-temperature equivalence; therefore, valid data that are outside the test frequency and temperature range can be obtained by means of calculation [51,52]. The time-temperature equivalence principle indicates that the frequency at a given test temperature can be altered along the logarithmic frequency axis by a displacement factor. Of these, the shift factor is only related to temperature, and can be calculated by the WLF equation, as shown in Equation (6):

(6) $$\log_{10}\alpha_{T}=\frac{-C_{1}\left(T-T_{0}\right)}{C_{2}+\left(T-T_{0}\right)}$$

where $\log_{10}\alpha_{T}$—shift factor; $C_{1}$,$C_{2}$—constants; T—test temperature, °C; $T_{0}$—reference temperature, °C.

**Figure 6:**
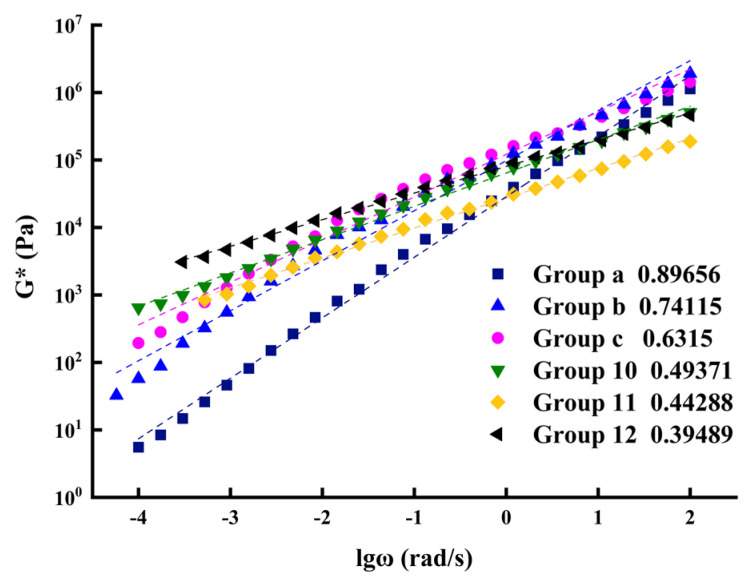
The linear fit curve of complex shear modulus (reference temperature: 30 °C).

Based on the time-temperature equivalence principle, the reference temperature was set as 30 °C, and the shift factors of six asphalt sealants at different temperatures were calculated according to Equation (5) (listed in Table 6), and the main curves of complex shear modulus of composites were drawn (shown in Figure 6). 

The slope of the curve obtained by linear fitting is able to reflect the variation trend of complex shear modulus with frequency. Therefore, this slope can be used to characterize the ratio of complex shear modulus to angular frequency. In Figure 6, it can be seen that the ratios of complex shear modulus and angular frequency of the six materials, in decreasing value, are in the following order: Group a > Group b > Group c > Group 10 > Group 11 > Group 12. This indicates that the susceptibility of the modified asphalt sealant to temperature following modification with a composite of SBS and CR was less than that of the modified asphalt sealant modified with a single additive (either SBS or CR), and the five modified asphalt sealant materials had greater temperature sensitivity than the unmodified asphalt sealant.

Simultaneously, in the low-frequency range, the complex shear modulus of the three kinds of composite-modified asphalt sealant (Group 10, Group 11, and Group 12) was greater than that of the unmodified asphalt sealant and the asphalt sealant modified with a single additive (Group b and Group c). However, in the high-frequency range, the complex shear modulus of the three kinds of composite-modified asphalt sealant was lower than that of the unmodified asphalt sealant and the asphalt sealant modified with a single additive. The low frequency of the primary curve corresponds to asphalt’s performance at high temperatures, while the high frequency refers to the asphalt’s low-temperature performance. Consequently, it can be inferred that the composite-modified asphalt sealant has greater resistance to permanent deformation than unmodified asphalt sealant and asphalt sealant modified with a single additive at elevated temperatures. Furthermore, the viscosity of the composite-modified asphalt sealant at low temperature was also superior to that of the unmodified asphalt sealant and the asphalt sealant modified with a single additive at low temperature.

In addition, in order to more clearly describe the change rule of the master curve, the CAM model was used to fit the master curves, and is expressed as shown in Equation (7):

(7) $$\left|G*\right|=\frac{\left|G_{g}*\right|}{{\left[1+f_{c}/{\left(\alpha_{T}f\right)}^{K}\right]}^{M/K}}$$

where $\left|G_{g}*\right|$ is the glassy shear modulus of the asphalt sealant; *K* and *M* are the shape-fitting parameters of the master curve;$\;f_{c}$ is the position-fitting parameter of the master curve; f is the loading frequency; and $\alpha_{T}$ is the shift factor.

**Figure 7:**
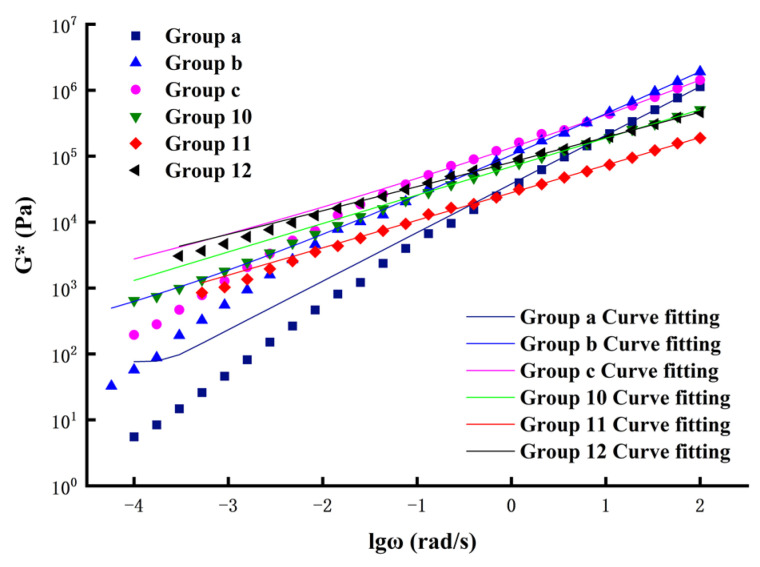
The main curve of complex shear modulus (reference temperature: 30 °C).

The fitting curves are shown in Figure 7. It can be observed that the CAM model has a high degree of fitting to the sample, indicating that the CAM model is suitable for fitting the dynamic modulus master curves of various sealants. In this way, the main curve of dynamic shear modulus was also drawn using the CAM model in order to verify that the CAM model is able to fit the main curves of various types of asphalt sealant, predict the changing trend of asphalt sealant shear modulus, and perform a reasonable evaluation of the rheological properties of modified asphalt sealants.

In summary, the mechanical properties of asphalt sealant materials are very sensitive to the frequency of load action. When the load frequency is low, the viscous properties of the asphalt sealant material are more obvious; when the load frequency in increased, the elastic properties of the asphalt sealant material become more obvious [51]. On the basis of the frequency sweep experiment, it can be seen that the modified asphalt sealant had lower temperature sensitivity, among which Group 12 had the lowest temperature sensitivity. Compared with the other groups of modified asphalt sealants, Group 12 still had better elasticity at high temperatures and better viscosity at low temperatures. This gives asphalt sealant modified with 20% CR and 7% SBS broad application prospects in cold regions.

## 4. Conclusions

With the aim of addressing the issue of the low resistance of conventional sealants to high-temperature deformation, this paper utilized waste rubber and SBS to prepare an eco-friendly asphalt sealant material for pavement maintenance engineering that possessed excellent high-temperature stability and anti-deformation ability, expanding the application of waste rubber in pavement maintenance engineering. Additionally, the high-temperature and rheological properties of this new type of SBS/CR-composite-modified asphalt sealant were estimated by performing a softening point test, a temperature scan test, a frequency scan test, and an MSCR and frequency sweep test. The following conclusions can be drawn:

(1) SBS and CR can improve the high-temperature performance of sealants, and the softening point of the modified sealant was able to meet the Chinese national standard (JT/T 740-2015). On the basis of grey correlation analysis, the correlation between SBS and the asphalt sealant softening point was 1.5 times that between CR and the asphalt sealant softening point.
(2) The temperature sweep test revealed that both SBS and CR can increase the complex shear modulus while simultaneously decreasing the phase angle, thereby improving the performance of the modified asphalts in high-temperature environments. In all groups, asphalt sealant modified with 20% CR and 5% SBS demonstrated superior deformation resistance and high-temperature resistance. The temperature sweep test revealed that SBS and CR improved the elasticity of the modified asphalt sealants in high-temperature environments. With comprehensive consideration of the composite shear modulus and phase angle of the sealants, the viscoelastic balance of the 20% CR and 5% SBS-modified asphalt sealant was more suitable for crack repair in all groups.
(3) The MSCR test showed that the composite modification procedure greatly improved the stiffness and elasticity of the modified asphalt sealants, among which the 20% CR and 7% SBS-modified asphalt sealants had the lowest $\bar{J_{nr}}$ and the highest $\bar{R}$, implying that it had good deformation resistance.
(4) The complex main curve of shear modulus for different asphalt sealant materials at 30 °C showed that the SBS/CR-composite-modified asphalt sealant possessed greater persistent deformation resistance at elevated temperatures and lower temperature sensitivity than unmodified asphalt sealant and asphalt sealant modified with a single modifier. Additionally, the CAM model demonstrated a good ability to fit to the dynamic model curve of the modified asphalt sealant and was able to be used to reasonably evaluate the rheological properties of the sealant under load conditions that are difficult to test.

This paper systematically evaluated the high-temperature rheological properties of CR/SBS-modified asphalt sealant. As for determining the actual environmental benefits and actual road performance of this new kind of asphalt sealant, this is a topic on which the authors will deliberate in the future.

## Figures and Tables

**Table 1:** Properties of 90# matrix asphalt.

Index	Standard Value	Measured Results
Softening Point (Global Method)	≥45 (°C)	46.0 (°C)
Penetration (25 °C, 100 g, s)	80~100 (0.01 mm)	84 (0.01 mm)
Ductility (15 °C)	≥100 (cm)	>100 (cm)
TFOT (Thin Film Oven Test) Residue		
Quality Change	±0.8 (%)	−0.112 (%)
Residual Penetration Ratio	≥57 (%)	62.4 (%)
Residual Ductility (10 °C)	≥8 (cm)	11.9 (cm)

**Table 2:** Technical indices of CR.

Index	Standard Value	Measured Value
Sieving rate	≥45%	91%
Ash content	≤10%	4.5%
Moisture content	˂1%	0.6%
Fiber content	˂1%	0.5%
Rubber content	≥48%	51%
Burn Participation	˂38%	37.5%

**Table 3:** The mix proportions for the CR- and SBS-compound-modified sealants.

Group	CR Contents (%)	SBS Contents (%)
1	10	1
2	10	3
3	10	5
4	10	7
5	15	1
6	15	3
7	15	5
8	15	7
9	20	1
10	20	3
11	20	5
12	20	7

**Table 4:** The difference sequences of CR and SBS.

Index	Sample											
	1	2	3	4	5	6	7	8	9	10	11	12
CR	10	10	10	10	15	15	15	15	20	20	20	20
SBS	1	3	5	7	1	3	5	7	1	3	5	7
Difference sequence	35.5	29.2	23	20.6	30	24.1	17.6	14.5	17.3	11.9	4.7	0

**Table 5:** The grey correlation coefficients for different contents of modifiers.

Factor	k												Grey Relation Coefficient
	1	2	3	4	5	6	7	8	9	10	11	12	
R1	0.64	0.64	0.64	0.64	0.54	0.54	0.54	0.54	0.47	0.47	0.47	0.47	0.55
R2	0.94	0.85	0.78	0.72	0.94	0.85	0.78	0.72	0.94	0.85	0.78	0.72	0.82

**Table 6:** Shift factors and C1 and C2 parameter values of materials at different test temperatures (reference temperature: 30 °C).

Asphalt	Parameter	Shift Factor						R^2^
	C1	C2	30	40	50	60	70	
Group a	6.349	46.509	0	−1.217	−1.844	−2.433	−2.990	0.9952
Group b	10.508	86.7	0	−1.045	−1.988	−2.732	−3.295	0.9993
Group c	9.329	87.64	0	−1.078	−1.642	−2.345	−2.964	0.9933
Group 10	10.109	100.127	0	−0.905	−1.718	−2.295	−2.899	0.9993
Group 11	6.188	42.558	0	−1.117	−2.002	−2.633	−2.944	0.9971
Group 12	17.545	238.033	0	−0.710	−1.301	−2.046	−2.491	0.9962

## Data Availability

The data presented in this study are available on request from the corresponding author.
